# FLAIR^2^ post-processing: improving MS lesion detection in standard MS imaging protocols

**DOI:** 10.1007/s00415-021-10833-x

**Published:** 2021-10-08

**Authors:** Tobias Zrzavy, Alice Wielandner, Lukas Haider, Sophie Bartsch, Fritz Leutmezer, Thomas Berger, Karl Heinz Nenning, Alexander Rauscher, Paulus Rommer, Gregor Kasprian

**Affiliations:** 1 Department of Neurology, Medical University of Vienna, Waehringer Guertel 18-20, 1090 Vienna, Austria; 2 Department of Biomedical Imaging and Image Guided Therapy, Medical University of Vienna, Vienna, Austria; 3 NMR Research UnitDepartment of NeuroinflammationFaculty of Brain Science, Queens Square MS CentreUCL Queen Square Institute of NeurologyUniversity College London, London, UK; 4 UBC MRI Research Centre, University of British Columbia, Vancouver, BC Canada

**Keywords:** MS, Cortical lesion, Imaging, Lesion detection, Post-processing

## Abstract

**Background:**

Technical improvements in magnetic resonance imaging (MRI) acquisition, such as higher field strength and optimized sequences, lead to better multiple sclerosis (MS) lesion detection and characterization. Multiplication of 3D-FLAIR with 3D-T2 sequences (FLAIR^2^) results in isovoxel images with increased contrast-to-noise ratio, increased white–gray-matter contrast, and improved MS lesion visualization without increasing MRI acquisition time. The current study aims to assess the potential of 3D-FLAIR^2^ in detecting cortical/leucocortical (LC), juxtacortical (JC), and white matter (WM) lesions.

**Objective:**

To compare lesion detection of 3D-FLAIR^2^ with state-of-the-art 3D-T2-FLAIR and 3D-T2-weighted images.

**Methods:**

We retrospectively analyzed MRI scans of thirteen MS patients, showing previously noted high cortical lesion load. Scans were acquired using a 3 T MRI scanner. WM, JC, and LC lesions were manually labeled and manually counted after randomization of 3D-T2, 3D-FLAIR, and 3D-FLAIR^2^ scans using the ITK-SNAP tool.

**Results:**

LC lesion visibility was significantly improved by 3D-FLAIR^2^ in comparison to 3D-FLAIR (4 vs 1; *p* = 0.018) and 3D-T2 (4 vs 1; *p* = 0.007). Comparing LC lesion detection in 3D-FLAIR^2^ vs. 3D-FLAIR, 3D-FLAIR^2^ detected on average 3.2 more cortical lesions (95% CI − 9.1 to 2.8). Comparing against 3D-T2, 3D-FLAIR^2^ detected on average 3.7 more LC lesions (95% CI 3.3–10.7).

**Conclusions:**

3D-FLAIR^2^ is an easily applicable time-sparing MR post-processing method to improve cortical lesion detection. Larger sampled studies are warranted to validate the sensitivity and specificity of 3D-FLAIR^2^.

**Supplementary Information:**

The online version contains supplementary material available at 10.1007/s00415-021-10833-x.

## Introduction

Multiple sclerosis (MS) is a chronic inflammatory demyelinating disease of the central nervous system (CNS) in which magnetic resonance imaging (MRI) is an invaluable diagnostic tool in establishing the diagnosis [22]. Improved lesion detection either by increasing field strength or improving acquisition and post-processing techniques possibly beneficially impacts time to diagnosis and disease monitoring [2, 25, 26].

While white matter (WM) lesions have long been the main focus of research in MS, cortical lesions (CL) are more and more recognized as playing an important role [12]. In the recent update of the diagnostic imaging criteria, the detection of cortical und juxtacortical (JC) lesions received additional weight [22]. In contrast to WM lesions, which are easily identified using standard MRI techniques [8], less than 25% of histopathologically confirmed CL are detectable by conventional (FLAIR and T2) clinical MR imaging [4]. Furthermore, both white and gray matter lesions have been associated with disability accumulation and potential outcome parameters in clinical trials [7, 10]. Therefore, there is a specific demand for high sensitivity in lesion detection of CL and JC lesions by means of MRI.

Current guidelines for standardized brain and spinal cord MRI for the diagnosis and follow-up of MS include 3D-T2-FLAIR and 3D-T2-weighted images [13, 23]. It was recently shown that the combination of 3D-T2 weighted images with 3D-FLAIR, referred to as 3D-FLAIR^2^, leads to a better contrast-to-noise ratio and white–gray-matter contrast, while still suppressing CSF signals and thereby to improved lesion visualization without the need for additional scan time [27].

In this monocentric study, we aimed to test the practicability of retrospectively computed 3D-FLAIR^2^ images of routinely acquired MRIs. We tried to prove or reject the hypothesis that 3D-FLAIR^2^ shows a higher sensitivity compared to 3D-T2-FLAIR or 3D-T2-weighted images alone in detecting cortical, LC, JC, and WM lesions in MS patients.

## Materials and methods

### Ethics

The study was approved by the ethics committee of the Medical University Vienna (ethical approval number: EK1464/2017).

### Patients and definitions

This is a retrospective analysis of patients recruited from the Department of Neurology, Medical University of Vienna between 2017 and 2018. The first thirteen MS patients from the dataset from the Vienna MS database (VMSD), with a high lesion load noted on previously addressed MRIs, were included in this study. All patients fulfilled the McDonald MS criteria [22].

### Image acquisition

MRI scans were acquired using a 3 T Achieva Philips Healthcare MRI scanner using an 8-channel SENSE head coil. 3D-FLAIR: voxel size = 0.67 mm × 0.76 mm × 1.34 mm; TR = 4800 ms; TE = 415 ms; TI = 1650 ms; Fat suppression: 3D-T2 = TR – 2500 ms, TE = 314 ms, acquired voxel size = 0.67 mm × 0.76 mm × 1.34 mm, reconstructed voxel size: 0.65 mm × 0.65 mm × 0.65 mm; SPIR; acquired voxel size = 0.67 mm × 0.76 mm × 1.34 mm; reconstructed voxel size = 0.65 mm × 0.65 mm × 0.65 mm; post sequences were reconstructed in three orthogonal planes.

Data processing was performed with Advanced Normalization Tools (ANTs v2.2.0) and FSL (v6.0). Both, T2 and FLAIR volumes were bias field corrected using N4 [24], and subsequently, image intensities were normalized. FLAIR images were co-registered and resampled to the T2 volume space using FLIRT [14, 15] (12 degrees of freedom, mutual information, sinc interpolation). Finally, the 3-DFLAIR^2^ image was obtained by multiplication of the aligned FLAIR and T2 volumes.

### Image analysis

Image analysis includes total white matter lesion counts of WM, JC, and cortical/LC lesions. Lesions were manually labeled by two trained raters (TZ, PR) with 5 and 10 years of experience in MS imaging, randomly and blinded in 3D-T2, 3D-FLAIR, and 3D-FLAIR^2^ with the ITK-SNAP tool [28]. In the case of disagreement, a senior neuroradiological rater with more than 15 years of experience in MS imaging was consulted (GK) to reach consensus. Lesions were defined according to previous literature [9].

### Statistics

Statistical analysis was performed using IBM SPSS 20.0.0 (SPSS Inc, Chicago, IL, USA) and R studio (Version 1.2.5033, RStudio, Inc.). The power analysis was based on previous data on WM/CL lesion subtype-specific frequencies in different stages of the disease in individuals with MS [12] (*p* value 0.05, power 80%) [20].

Normal distribution was assessed and rejected for lesion count variables with Shapiro–Wilk’s method, which are provided with median value and interquartile range. Differences between two groups were assessed with the Mann–Whitney *U* test. Bland–Altman plots were calculated to quantify the amount of agreement in lesion counts derived from different sequences (3D-FLAIR^2^, 3D-FLAIR, and 3D-T2) and different raters (TZ vs. PR TZ vs. TZ) for WM, JC, and LC lesions. The mean difference and the limits of agreement, which reflect the 95% level as described by Bland and Altman [3] are provided with their 95% CI.

Inter- and intra-rater variability was assessed with intraclass correlation coefficient for all sequences using a two-way random-effects model with absolute agreement [17]. Intra-rater variability was assessed by two independent lesion counts by rater TZ (TZ1 vs. TZ2), while inter-rater variability was assessed by calculating lesion assessment of (TZ1 vs PR1). Significance was set at a two-sided *p* value of 0.05.

### Data availability

Anonymized data not published in the article can be made available upon reasonable request from a qualified investigator after approval from the ethics review board of the Medical University of Vienna.

## Results

### Patient characteristics

13 patients with a confirmed diagnosis of MS [3 males and 10 females, mean age 37.3 years ± 13.9 (SD)] were included. The median EDSS score was 2.5; 4.5 (IQR). The mean disease duration was 6.3 years ± 6.0 (SD). Nine patients were classified as having a relapsing remittent disease course, three patients presented a secondary progressive and one patient with a primary progressive disease course.

### Lesion detection

A total of 1067 3D-FLAIR^2^, 809 3D-FLAIR, and 577 3D-T2-weighted lesions were detected in these 13 patients.

**Fig. 1:**
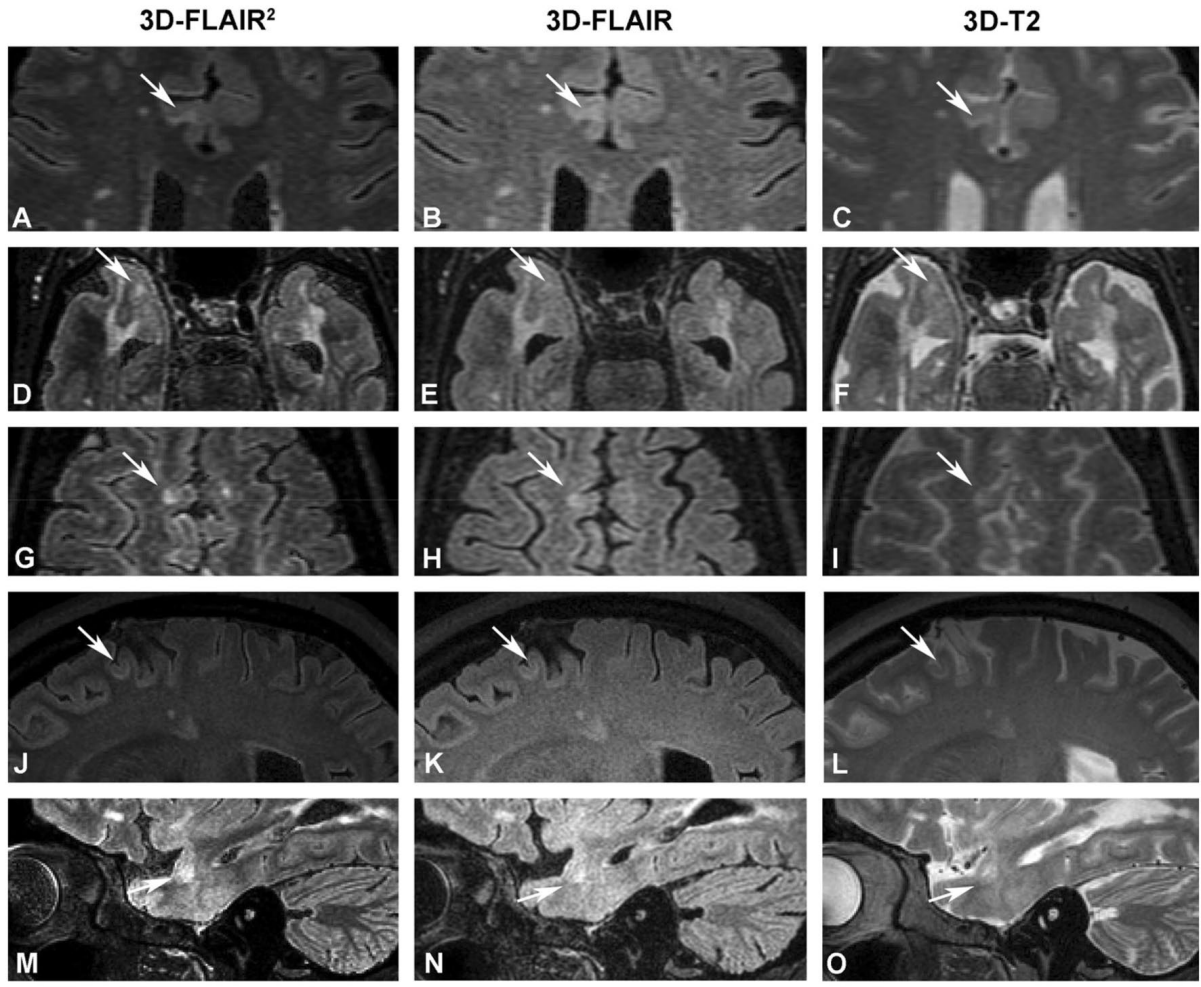
3D-FLAIR^2^ lesion visualization compared to 3D-T2 and 3D-FLAIR. A-C: Depiction of a cortical/leucocortical lesion on axial 3D-FlAIR^2^ (**A**), 3D-FLAIR (**B**), 3D-T2 (**C**) MRI images. **D**–**F** Presentation of a large white matter lesion with an adjacent cortical/leucocortical lesion. **G**–**I** Juxtacortical lesion involving the right frontal superior gyrus; **J**–**L** Juxtacortical U-fiber lesion on sagittal view. **M**–**O** Temporal cortical/leucocortical lesion on sagittal view

**Fig. 2:**
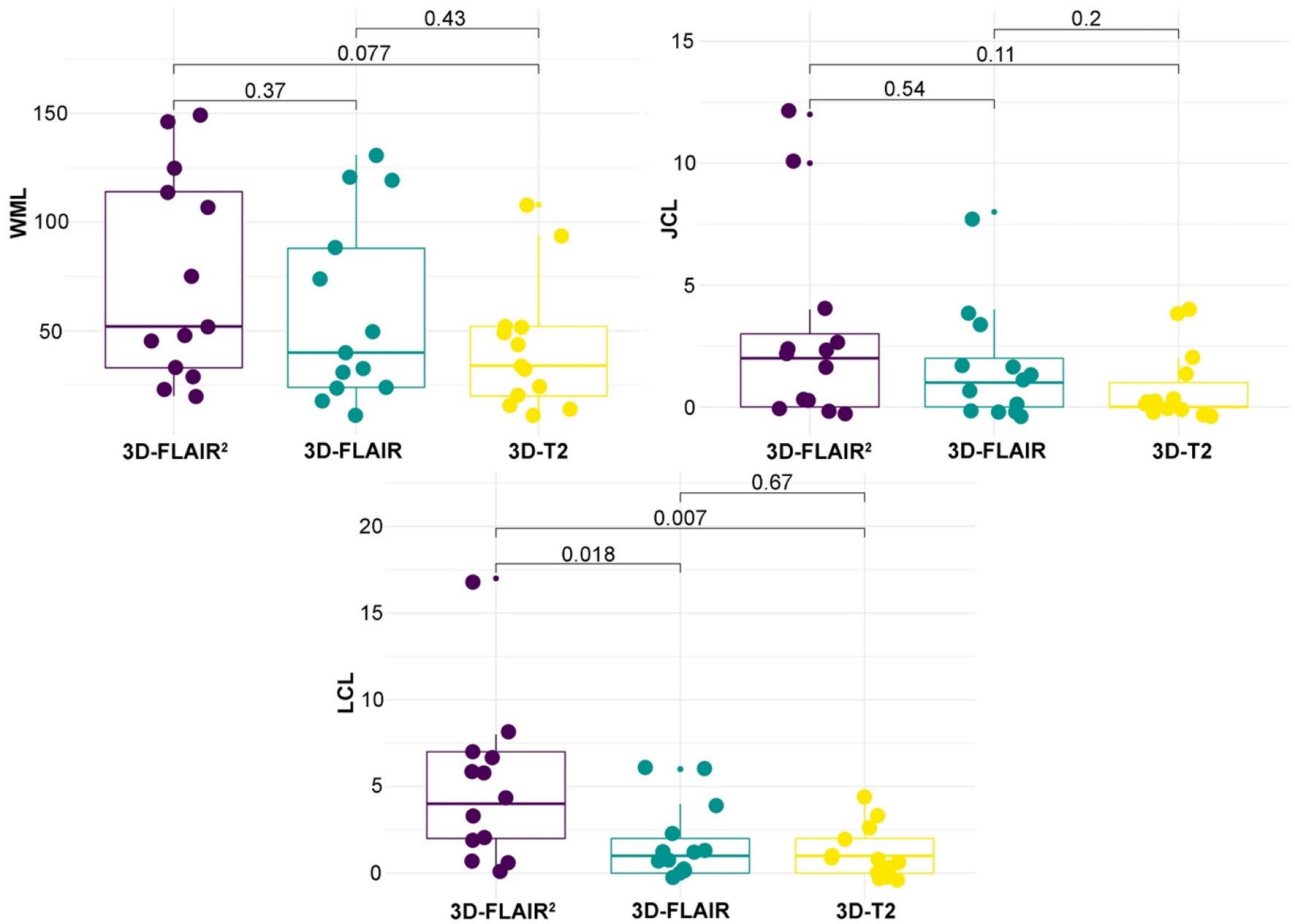
Quantitative lesion evaluation in 3D-FLAIR2 in comparison to 3D-FLAIR and to 3D-T2. *LCL* Leucocortical lesion, *JCL* Juxtacortical lesion, *WML* White matter lesion

WM lesions were more common on 3D-FLAIR^2^ sequences compared to 3D-FLAIR (median 52 vs 40, *p* = 0.37) and 3D-T2-weighted scans (median 52 vs 34, *p* = 0.077) (Figs. 1, 2).

Overall, 70 JC were counted. In 3D-FLAIR^2^ images more JC lesions were counted compared to 3D-FLAIR (median 2 vs 1; *p* = 0.54) and 3D-T2-weighted images (median 2 vs 0; *p* = 0.11), however not reaching statistically significance.

**Fig. 3:**
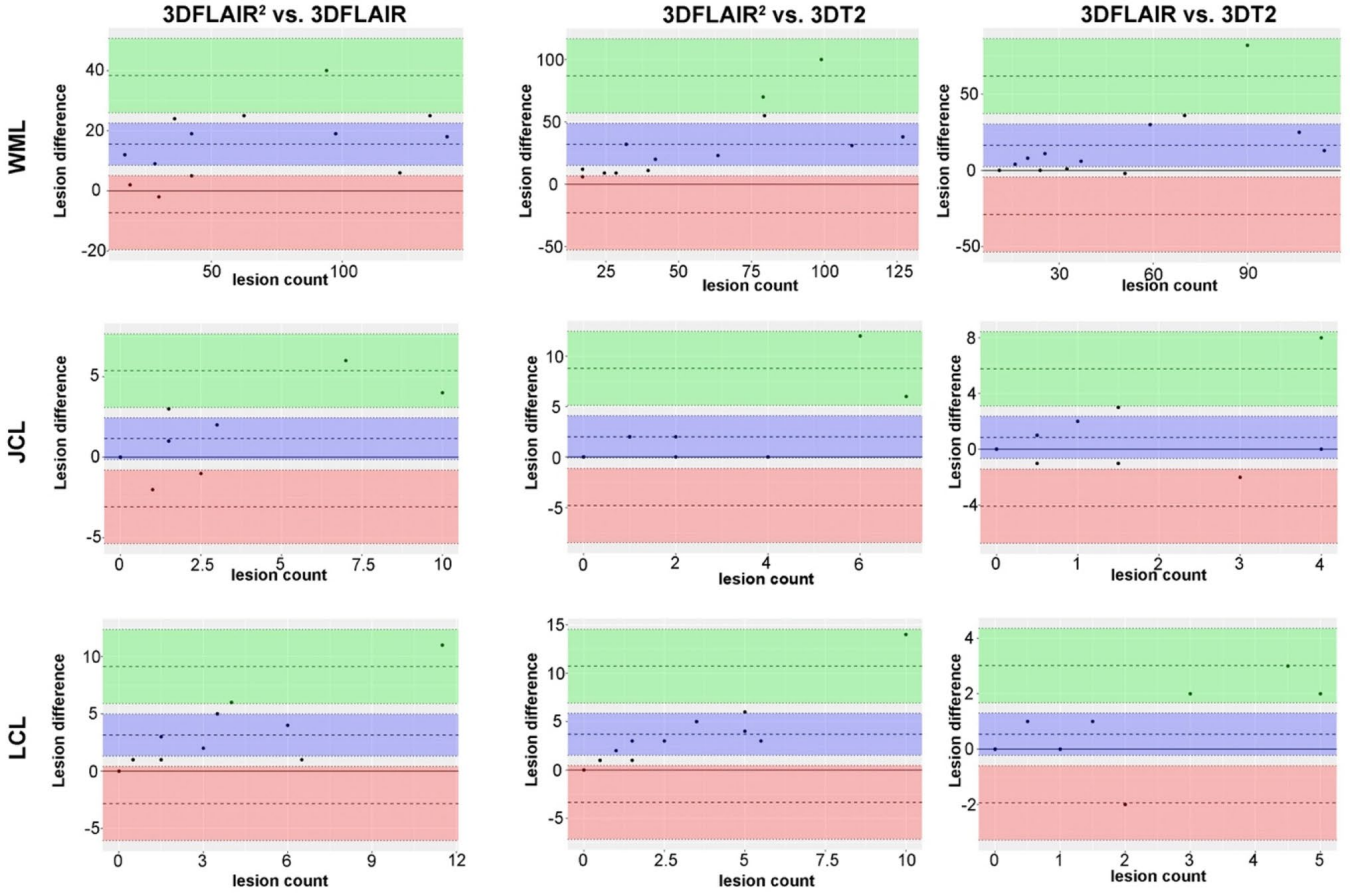
Difference in the numbers of assessed lesions. Bland–Altman plots comparing lesion counts derived from 3D-FLAIR^2^, 3D-FLAIR and 3D-T2 in each patient and each location. For white matter lesion (WML), juxtacortical lesion (JCL) and leucocortical lesion (LCL), the difference between lesion counts derived from the different sequences (3D-FLAIR^2^, 3D-FLAIR and T2), is plotted relative to their mean for each patient with black dots. The black dashed line provides the mean difference with the corresponding 95% CI in blue. The limits of agreement are provided with their 95% CI, the upper bound in green, the lower bound in red

LC lesion visibility was significantly improved by 3D-FLAIR^2^ in comparison to 3D-FLAIR (median 4 vs 1; *p* = 0.018) and 3D-T2 (median: 4 vs 1; *p* = 0.007). Comparing LC lesion detection in 3D-FLAIR^2^ vs 3D-FLAIR, 3D-FLAIR^2^ detected on average 3.2 more cortical lesions (95% CI − 9.1 to 2.8) per patient. Comparing against 3D-T2, 3D-FLAIR^2^ detected on average 3.7 more LC lesions (95% CI 3.3–10.7) (Fig. 3).

**Fig. 4:**
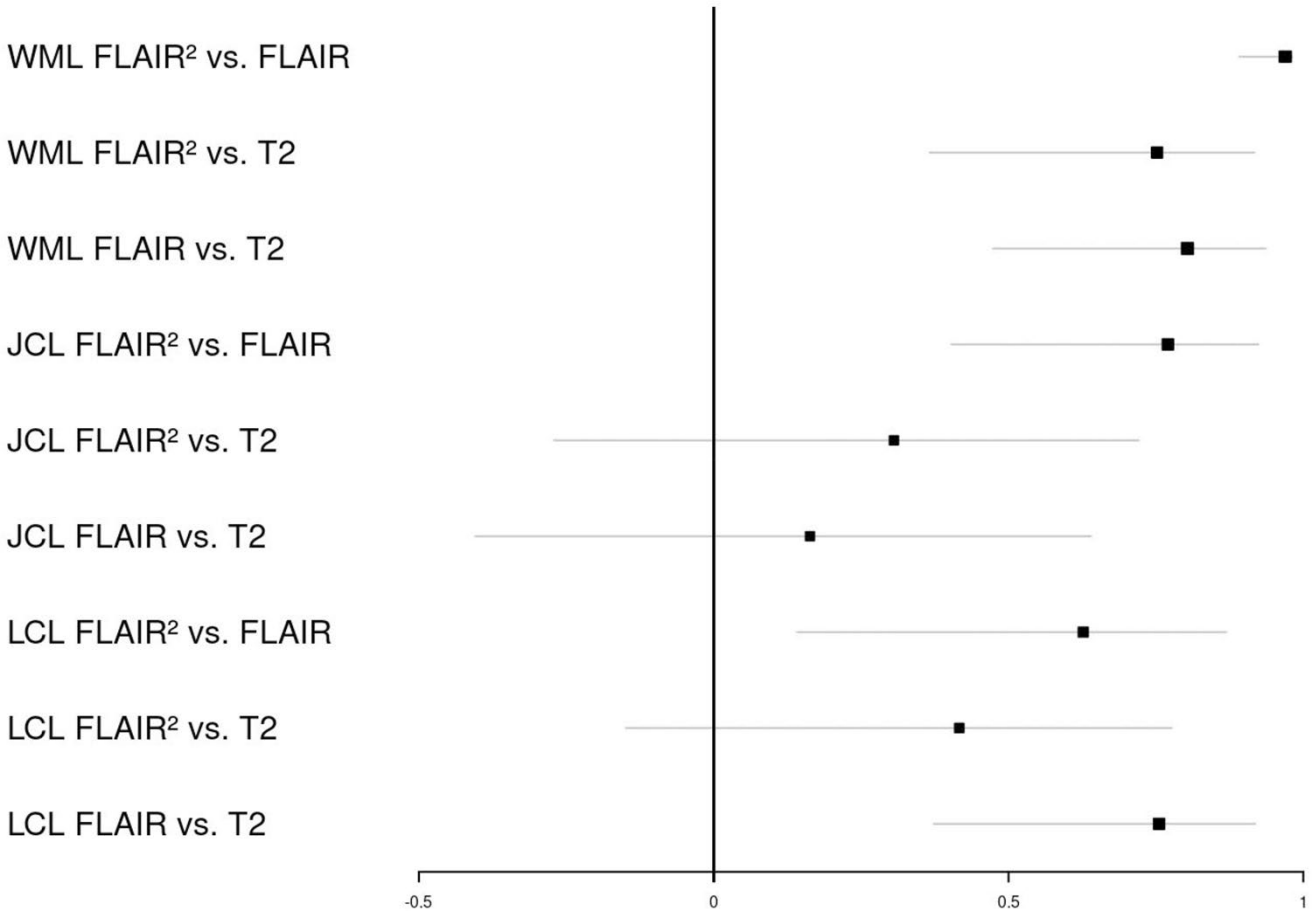
Interclass correlation for 3D-FLAIR^2^, 3D-T2 and 3D-FLAIR. ICC provided with their 95%CI (gray); *WML* white matter lesion, *JCL* juxtacortical lesion, *LCL* leucocortical lesion

We further calculated the interclass correlation coefficients (ICC) to quantify the amount of correlation adjusted for random effects between lesion counts obtained from different sequences (Fig. 4). The highest ICC was calculated for WM lesion followed by those from JC and LC lesion metrics.

JC lesions measured on 3D-T2 vs 3D-FLAIR^2^ (0.30 ICC, 95% CI − 0.27 to 0.72) or 3D-FLAIR (0.16 ICC, 95% CI − 0.40 to 0.64) and LC lesions in 3D-T2 vs 3D-FLAIR^2^ (0.41 ICC, 95% CI − 0.15 to 0.78) displayed a 95% CI involving zero.

To determine the reproducibility for 3D-FLAIR^2^ intra-rater and intra-correlation coefficients were calculated 0.90 (WM), 0.88 (JC) and 0.86 (LC). Interrater intra-correlation for 3D-FLAIR^2^ were 0.91 (WM), 0.78 (JC) and 0.75 (LC) (Suppl. Figure 1/Suppl Table 1).

## Discussion

Detection of lesions by MRI is an integral component of both diagnosis and disease monitoring in MS [22, 23]. Here, we assessed the added diagnostic value of a voxel-wise multiplication of 3D-T2 weighted images with 3D-FLAIR images, resulting in 3D-FLAIR^2^ compared to the acquired initially standard 3D sequences alone. We demonstrated that 3D-FLAIR^2^ increases the detection rate of LC lesions compared with state-of-the-art T2 and FLAIR 3D sequences.

The potential benefit of 3D-FLAIR^2^ was previously shown by demonstrating a higher contrast-to-noise ratio for WM and GM lesions in comparison to FLAIR or T2 images [27]. It was proposed that this approach produces a similar contrast like double inversion recovery (DIR), however, with improved image quality and less acquisition time. While FLAIR^2^ was first proposed with 3D scans, it can also be used with 2D-FLAIR and 2D-T2 scans [18]

In line with these data, we provide evidence that 3D-FLAIR^2^ outperformed state-of-the-art 3D-FLAIR and 3D-T2-weighted sequences in lesion visualization of cortical lesions without the need for additional image acquisition time. 3D-FLAIR^2^ is a sensitive and radiologically feasible tool in clinical routine and clinical studies for MS lesion assessment at not cost of imaging time. As 3D-FLAIR^2^ may also improve automatic lesion segmentation, it could easily be implemented in future automatic MS lesion detection algorithms [18]. Automatized segmentation and Artificial intelligence (AI) of MRI images have great potential in monitoring disease activity in demyelinating diseases of the central nervous system and guiding diagnostic pathways [1, 16, 19], 3D-FLAIR^2^ may further improve the quality of diagnosing and monitoring these patients non-invasively. Likewise, it will also increase human capabilities in lesion detection and potentially help in the supervision of machine learning.

As WM lesion load only in part explains clinical disease progression, conversion and cognitive decline, cortical lesions increasingly become a focus of research [5]. Despite advances in imaging, even under optimal conditions, a maximum of 25% of the actual dimension can be visualized in histopathology correlation studies [4]. Improving cortical lesion detection has the potential to improve the prediction of subsequent disease evolution and therapeutic response and as well as to improve fulfilling the criterion of dissemination in space [6, 22]. Here, we could show that 3D-FLAIR^2^ can enhance the detection of cortical/leucocortical lesions compared to standard routine sequences and display excellent inter and intra-rater variability (Figs. 3,4).

It should be noted that our sample consists of preselected MS patients with previously noted high cortical lesion load. Further limitations of this study include its retrospective nature. We used 3D images as reference images for comparison since they outperform lesion detection compared to 2D images [11, 21]. We could not compare our findings to PSIR and DIR images, as these are not part of the used MS MRI protocols. Therefore, future prospective studies should include a larger number of subjects, potential histopathological correlations, and direct comparison with DIR/PSIR to determine the value of 3D-FLAIR^2^ in improving disease monitoring and MS diagnosis.

In summary, we show that combining 3D-T2 and 3D-FLAIR sequence data to create 3D-FLAIR^2^ is a feasible and easily applicable strategy to specifically improve cortical/leucocortical lesion detection in MS.

## Supplementary Information

Below is the link to the electronic supplementary material.

Supplementary file1 (DOCX 439 KB)
