# Correction to: FLAIR^2^ post-processing: improving MS lesion detection in standard MS imaging protocols

**DOI:** 10.1007/s00415-021-10855-5

**Published:** 2021-10-26

**Authors:** Tobias Zrzavy, Alice Wielandner, Lukas Haider, Sophie Bartsch, Fritz Leutmezer, Thomas Berger, Karl Heinz Nenning, Alexander Rauscher, Paulus Rommer, Gregor Kasprian

**Affiliations:** 1 Department of Neurology, Medical University of Vienna, Waehringer Guertel 18-20, 1090 Vienna, Austria; 2 Department of Biomedical Imaging and Image Guided Therapy, Medical University of Vienna, Vienna, Austria; 3 NMR Research UnitDepartment of NeuroinflammationFaculty of Brain Science, Queens Square MS CentreUCL Queen Square Institute of NeurologyUniversity College London, London, UK; 4 UBC MRI Research Centre, University of British Columbia, Vancouver, BC Canada

## Correction to: Journal of Neurology 10.1007/s00415-021-10833-x

In the original version of this article, the given and family names were incorrectly structured.

The correct given and family names should be:

Tobias Zrzavy

Alice Wielandner

Lukas Haider

Sophie Bartsch

Fritz Leutmezer

Thomas Berger

Karl Heinz Nenning

Alexander Rauscher

Paulus Rommer

Gregor Kasprian

The original article has been corrected.

