# Supplementary material for: FLAIR2 post-processing: improving MS lesion detection in standard MS imaging protocols
Source: J Neurol. 2021 Oct 8;269(1):461–7. doi: 10.1007/s00415-021-10833-x (PMC8738502; doi:10.1007/s00415-021-10833-x)
Supplement: Supplementary file 1 — Supplementary file1 (DOCX 439 KB) [file 415_2021_10833_MOESM1_ESM.docx]

**Supplementary Figure**


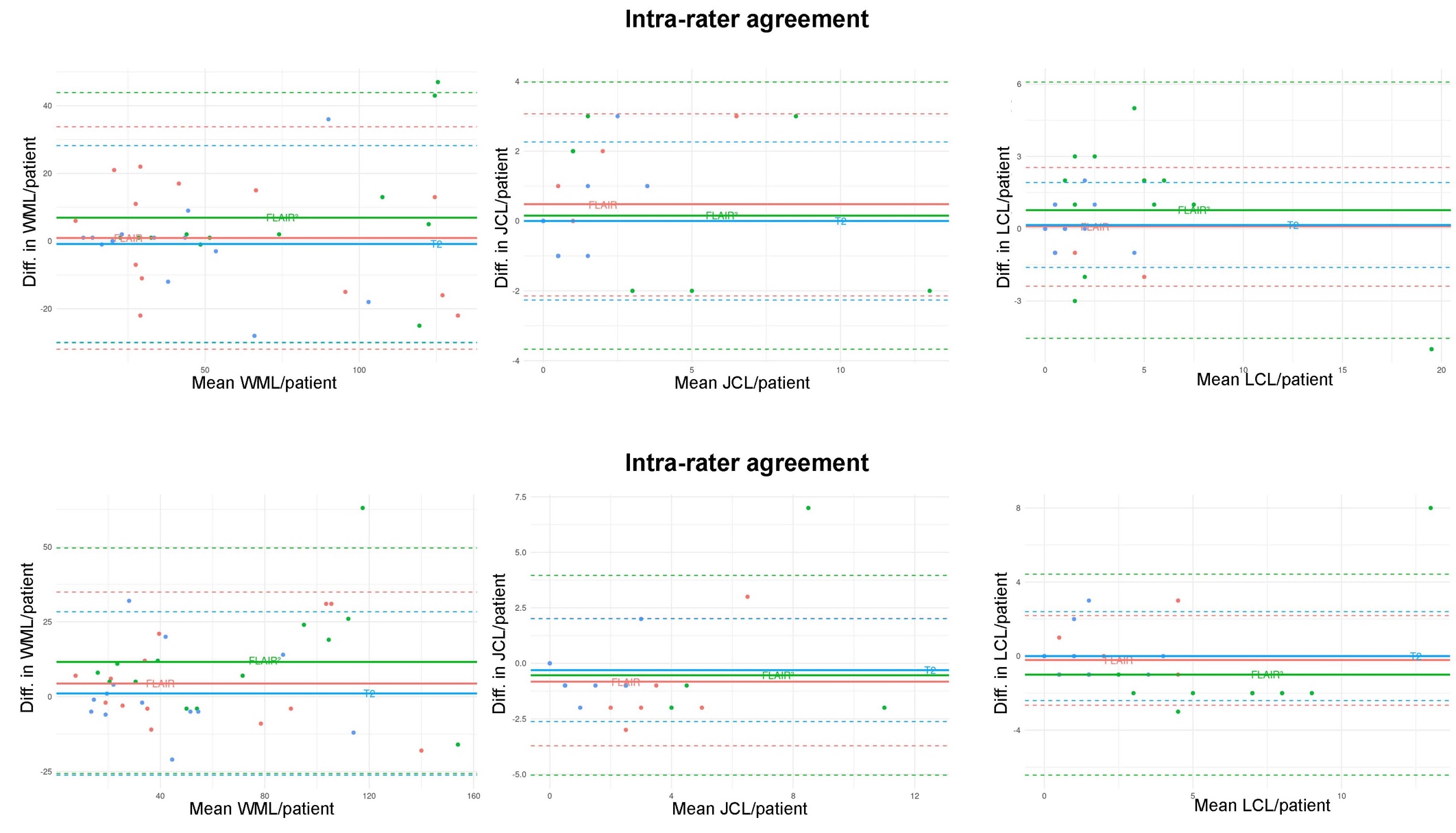
 WML: White matter lesion, JCL: juxtacortical lesion, LCL leucocortical lesion, average difference (thick red/green/blue) and the limits of agreement are indicated by dashed lines (red/green/blue), 95%CI for the estimates were not plotted in order to maintain readability.

**Supplementary Table 1**

| **intra-rater ICC** | | | | |
| --- | --- | --- | --- | --- |
| sequence | ICC | lower CI | upper CI | p-value |
| FLAIR_WML | 0,934 | 0,799 | 0,979 | 5,22E-07 |
| FLAIR_JCL | 0,781 | 0,426 | 0,928 | 4,84E-04 |
| FLAIR_LCL | 0,805 | 0,477 | 0,936 | 2,56E-04 |
| FLAIR2_WML | 0,908 | 0,726 | 0,971 | 3,66E-06 |
| FLAIR2_JCL | 0,882 | 0,658 | 0,962 | 1,52E-05 |
| FLAIR2_LCL | 0,863 | 0,611 | 0,956 | 3,59E-05 |
| T2_WML | 0,878 | 0,648 | 0,961 | 1,85E-05 |
| T2_JCL | 0,572 | 0,057 | 0,847 | 1,63E-02 |
| T2_LCL | 0,786 | 0,435 | 0,929 | 4,33E-04 |
|  |  |  |  |  |
| **inter-rater ICC** | | | | |
| sequence | ICC | lower CI | upper CI | p-value |
| FLAIR_WML | 0,933 | 0,795 | 0,979 | 5,86E-07 |
| FLAIR_JCL | 0,771 | 0,404 | 0,924 | 6,29E-04 |
| FLAIR_LCL | 0,845 | 0,569 | 0,950 | 7,04E-05 |
| FLAIR2_WML | 0,910 | 0,733 | 0,972 | 3,09E-06 |
| FLAIR2_JCL | 0,783 | 0,429 | 0,928 | 4,69E-04 |
| FLAIR2_LCL | 0,747 | 0,357 | 0,915 | 1,07E-03 |
| T2_WML | 0,897 | 0,699 | 0,968 | 6,72E-06 |
| T2_JCL | 0,574 | 0,060 | 0,848 | 1,59E-02 |
| T2_LCL | 0,630 | 0,146 | 0,870 | 7,90E-03 |

CI: confidence interval, ICC: interclass correlation coefficient; JCL: juxtacortical lesion, LCL leucocortical lesion, WML: White matter lesion
